# Periodic Table of Virus Capsids: Implications for Natural Selection and Design

**DOI:** 10.1371/journal.pone.0009423

**Published:** 2010-03-04

**Authors:** Ranjan V. Mannige, Charles L. Brooks

**Affiliations:** 1 Department of Chemistry and Biophysics Program, University of Michigan, Ann Arbor, Michigan, United States of America; 2 Center for Theoretical Biological Physics, University of California San Diego, La Jolla, California, United States of America; 3 Department of Molecular Biology, The Scripps Research Institute, La Jolla, California, United States of America; The Scripps Research Institute, United States of America

## Abstract

**Background:**

For survival, most natural viruses depend upon the existence of spherical capsids: protective shells of various sizes composed of protein subunits. So far, general evolutionary pressures shaping capsid design have remained elusive, even though an understanding of such properties may help in rationally impeding the virus life cycle and designing efficient nano-assemblies.

**Principal Findings:**

This report uncovers an unprecedented and species-independent evolutionary pressure on virus capsids, based on the the notion that the simplest capsid designs (or those capsids with the lowest “hexamer complexity”, 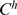) are the fittest, which was shown to be true for all available virus capsids. The theories result in a physically meaningful periodic table of virus capsids that uncovers strong and overarching evolutionary pressures, while also offering geometric explanations to other capsid properties (rigidity, pleomorphy, auxiliary requirements, etc.) that were previously considered to be unrelatable properties of the individual virus.

**Significance:**

Apart from describing a universal rule for virus capsid evolution, our work (especially the periodic table) provides a language with which highly diverse virus capsids, unified only by geometry, may be described and related to each other. Finally, the available virus structure databases and other published data reiterate the predicted geometry-derived rules, reinforcing the role of geometry in the natural selection and design of virus capsids.

## Introduction

Viruses are pervasive pathogens that infect organisms belonging to all domains of life [1]. A large number of these viruses (and their genomes) are enclosed and protected by spherical capsids–symmetric coats or shells composed primarily of multiple copies of protein subunits [2], [3]. Aside from serving as a protective layer, capsids are involved with various other aspects of their respective virus life cycles including timely viral genome encapsulation (self assembly and genome packaging), cell-to-cell virus transport, entry into host-cell (e.g., via cell receptor binding), genome release into host cell, etc. [3] Despite their central importance to the life cycle, the various evolutionary pressures acting on spherical capsids are not well known. In this report, we use theory to shed light on what seems to be an elusive but systematic and strong selection pressure on the various capsid sizes potentially available in nature.

Half a century of empirical data has uncovered a large array of capsid sizes that range from tens to many thousands in subunit composition [4]. Still, some sizes are rarer than others (those emboldened in Table S1 in File S1), an observation that puzzled structural virologists as early as 1961 [5], [6]. The cause for this discrepancy remains unexplained. Why are some capsid sizes not seen even today? Are specific spherical viruses disadvantaged from an evolutionary perspective? Or have we just not looked enough or in the right places? In this report, we present a conceptual framework useful in providing answers to these questions, while arriving at interesting observations about capsid classes, distributions, morphologies and mechanical properties. We first touch on useful concepts that lead to a capsid classification that is finally useful in developing the conclusions and schematic of this report.

**Figure 1:**
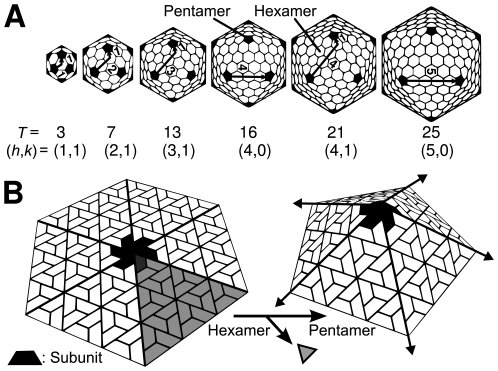
Capsids are scalable. (**A**) Spherical capsids of various sizes are composed of 12 pentamers (represented as darkened pentagons) and a variable number of hexamers. (**B**) Quasi-equivalence [6] posits that one may produce a pentamer from a hexamer by removing one subunit and its environment (the shaded triangular region) and joining the unpaired interfaces. This operation imposes pentameric dihedral angle values (“endo angles”) onto its neighboring hexameric angles [11], which, if unchallenged, propagate through the hexamers (depicted by arrows) in what we call *endo angle propagation*.

Spherical capsids of all observed sizes may be obtained from a grouping of twelve pentamers (symmetric clusters of five subunits) separated by a variable number of hexamers (clusters of six subunits) [5], [6] represented in Fig. 1A (as a diversion, more strictly speaking, the notion of the hexamer and pentamer must be replaced with hexavalent subunit clusters and pentavalent subunit clusters, respectively [6]. This is the case for the 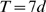 papillomaviruses [7] where all capsomers are made up of five subunits [but they are in both hexavalent and pentavalent configuration], and larger viruses whose “hexamers” are actually trimers of “fused” or covalently bonded dimers [8]).

Capsid size may be characterized by two integers, 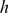 and 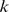 (first discussed by Goldberg [9]), which describe the number of hexamers (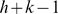) one would have to “walk over” to get from one pentamer to an adjacent pentamer within a completed capsid (the walk is shown as arrows in Fig. 1A) [6]. As a rule, a longer “walk” indicates the presence of more hexamers in the structure, which means a larger capsid. A useful metric for capsid size–the *triangulation number*, 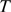 (where 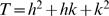)–was also introduced [6]; this number is useful because, in most cases, a capsid of triangulation number 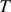 is comprised of 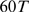 subunits, or 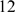 pentamers and 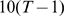 hexamers, i.e., 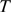 is a quantitative metric for capsid size. We now show, using “endo angles”, that 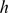 and 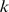 (and not 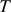) are sufficient in providing a useful capsid classification schematic.

## Results and Discussion

First, we will use the concept of the *endo angle constraint* to draw connections between a capsid classification scheme (developed below) and hexamer shapes present within a capsid. These concepts will then allow us to arrive at a metric for capsid complexity (hexamer complexity), which is useful in explaining and predicting various structural and evolutionary properties of the capsid.

### Endo Angles Classify Capsids

**Figure 2:**
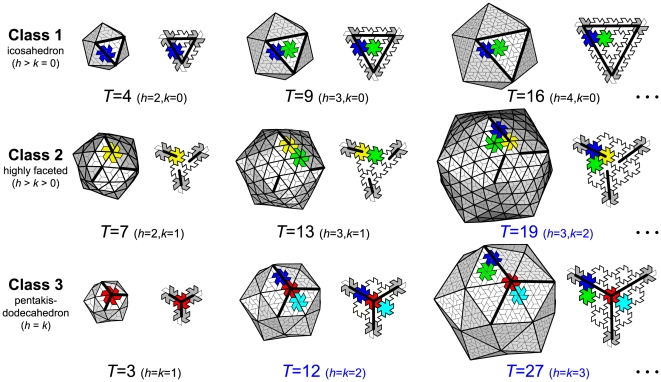
The three virus capsid classes. All canonical capsids (made up of trapezoidal subunits) may be built from a single type of pentamer and a repertoire of distinct hexamer shapes (colored distinctly only once in each capsid; also described in Fig. S2 in File S1). The hexamer shape is described by the number of endo angles it displays. Endo angles are depicted as bold lines within a “face” in its isolated (right) and capsid environment (left) for the first three capsid sizes in each class (excepting 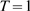).

The tilable nature of virus capsids [10] has uncovered a novel constraint on hexamers called *endo angle propagation* (it is a constraint imposed by pentamers onto hexamers; see Fig. 1B) that was crucial in predicting the existence of various distinct hexamer shapes [11]; here, hexamer shape is defined by the hexamer pucker or subunit-subunit planar angles within the hexamer (The number of hexamer shapes available are enumerated in Fig. S2 in File S1). In Section A of File S1, we show that there are *three* distinct distributions of endo angle patterns within a capsid (Fig. S1 in File S1), which ensures the emergence of three general morphological classes (Fig. 2 and Table S1 in File S1) differentiated by their 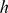-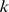 relationship: class 1 (described by the relationship 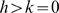), class 2 (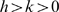), and class 3 (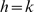). We will henceforth assume that 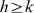 for simplicity's sake, since, for our discussions, the difference between chiral 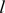 and 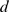 class 2 capsids is inconsequential.

As as brief but relevant detour, it is interesting that the classification system presented here was previously qualitatively recognized in the early 1960's to explain absentees in the capsid size diversity or 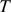-range (*Fig. 10* in Ref. [5] and *Fig. 8* in Ref. [6]; although both accounts did not directly link 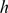 and 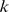 to class type). Specifically, class 2 capsids (in our schematic) had not yet been observed, and both reports postulated that capsids from this class must be absent for specific (but distinct) physical reasonings [5], [6]. Since then, capsids from all three classes have amply been seen (abundances are reported in Table S1 in File S1), i.e., the classification system can not be used to make direct predictions about capsid existence. Consequently, this topic, which we are readdressing now, appears to have been latent since 1962.

### Introducing Hexamer Complexity (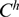)

The utility of the class system is not entirely lost, however; specific endo angle patterns within the capsid ensures the existence of distinct hexamer shapes (each shape is colored distinctly in Fig. 2). Here, we introduce the *hexamer complexity* (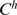) as the number of distinct hexamer *shapes* present in a capsid (a higher number of distinct hexamer shapes per capsid reflects a higher 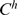). One may obtain 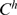 by counting the number of distinctly colored (shaped) hexamers in Fig. 2 (Note that in our schematic, hexamers in distinct environments are allowed to possess the same shape). We reason that capsids with higher 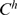 are evolutionarily disfavored.

### Using Hexamer Complexity as a Metric for Understanding Capsid Selection Pressures

#### High 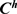 capsids require more auxiliary control during formation

Evidence indicates that capsid formation is nucleated [12], often starting with a single capsomer species (e.g., pentamers [13]; for the purposes of this paper, a capsomer is a generally symmetric cluster of either five or six subunits), which then proceeds to completion by the addition of small subunit clusters (or single subunits). In 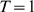 capsids, where subunits are in identical/equivalent environments [6], nucleated assembly will be possible with no additional machinery (except for the predefined angle of incidence for each subunit-subunit interaction site). However, the formation of two or more capsomers from a single interaction site will require the employment of additional machinery to ensure high yields of the native state. For example, quasi-equivalent switches [14], [15] are required for the proper assembly of capsids containing two distinct capsomers–a pentamer and one type of hexamer (i.e. 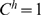). The addition of a second hexamer shape (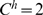) necessitates the requirement of a second mechanism such as auxiliary proteins [16] for proper assembly (discussed earlier in theory [11] and evidenced from the observation that all recorded 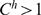 or 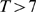 capsids are known to require auxiliary proteins for assembly [14]).

#### Capsid 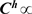 1/capsid abundance

For spherical virus capsids requiring more distinct hexamer shapes (larger 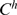), additional mechanisms to stabilize those new shapes at exactly the right positions within the forming capsid are likely to be also needed (lest off-pathway and fatal configurations would dominantly form), the interplay of which, we propose, would be theoretically possible to choreograph but unduly complex. Accordingly, we predict that canonical capsids with larger 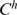 will be encountered with a lower frequency in nature (it is beyond any doubt that complexity is often not the sole criterion for natural selection. In fact, if that was the case then humans would never be given the chance to come into existence. But alongside natural selection arises the notion of the niche, that states that, among organisms that live within a niche and that compete for the same natural resources, the most efficient design will likely prevail. This comes into play when we consider spherical viruses that are dissimilar in 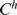 but operate under identical host and reproductive constraints. In those situations, the capsid with a simpler and more efficient design, i.e., those with low 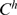, will be more efficient than the higher 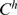 capsid in assembling, and therefore propagating).

**Figure 3:**
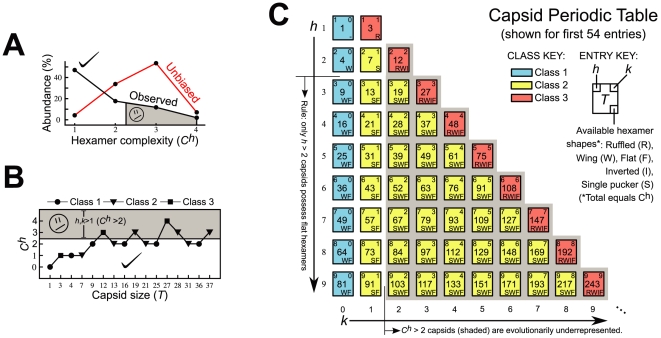
Periodic discrimination of spherical capsids. (**A**) As predicted by the inverse 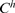 rule, capsids with high hexamer complexity are under-represented in nature as evident in the observed versus unbiased capsid abundances (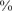 of families that display capids of specific 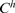). (**B**) 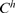 is not conveniently correlated with capsid size (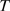) or class (symbols). (**C**) However, trends in 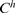 are easily discerned from the periodic table, where, in each period (row), 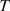, class number and 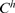 increase (or remain the same), while trends in other capsid properties such as rigidity may also be deciphered.

Support for this relationship (that high 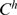 will be encountered with lower frequency in nature) is presented in Fig. 3A (and discussed further in Fig. S3 in File S1), where there is an inverse correlation between capsid 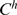 (calculated using Eqn. 1) and observed capsid abundance (for 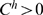 capsids, listed in Section L in File S1, were pooled from EM and X-ray structure repositories [4], [17]. We did not distinguish between capsids containing external lipid membranes and those that do not, since, often, such lipids are post assembly features [18]). However, this is not the case for unbiased capsid distributions (red line) where we assume no evolutionary favoritism (i.e., if we assume that each capsid size or 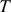 is equally probable to exist for the size range observed; 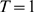 through 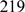). Also apparent in this data is the observation that 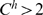 capsids are under-represented by a factor of 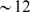 (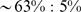 for unbiased vs. observed capsid abundances) when compared to the calculated distributions for the observed size range (if we calculate expected distributions for a more conservative range of 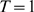 through 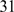, the unbiased value is still 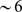 times higher than our observed 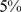 at 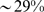). This suggests that a large evolutionary pressure in aversion to high hexamer complexity may be at play in nature.

#### Capsid 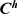 is related to class (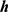,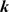) not size (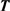)

Although not directly relatable to capsid size (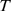) and class (Fig. 3B), 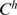 is easily obtained from the Goldberg parameters 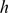 and 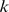 (Eqn. 1) from which we can show that 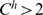 when both 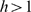 and 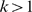 (Table S2 in File S1). 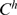 rules are concisely reiterated in periodic form in Fig. 3C such that, through each period (row), hexamer complexity (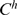), class number, and triangulation number (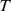) increase from left to right, allowing us to predict that capsids belonging to the right side of this table (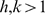) are evolutionarily disfavored (note that there is no one-to-one mapping of 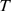 on 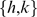; e.g., 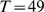 may be constructed from 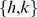 pairs 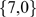 and 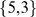 assigned to classes 1 and 2 respectively, i.e., some 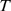 numbers will be repeated in the periodic table). Since capsid class describes distinct geometries, we expect that this table will also be useful in describing physical properties such as capsid rigidity.

Our complexity rules, although arising from geometric analysis of *canonical* capsid models [11] (further discussed in Sections A–D in File S1), appear to be applicable to almost all observed capsids, indicating that hexamer complexity may be a universally important concept (if we include only canonical capsids [10], the number of 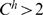 capsids reduce to zero!). We will shortly discuss the few “rule breakers”.

#### Designability vs. ease of construction

At this point, it is important to distinguish design from evolution. From a design perspective, capsids of any size (or 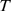 number) may be easily “built” from an intricate set of rules, like in a Lego® construction kit, i.e. capsids of any 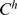 are viable designs. However, we suggest that, from an evolutionary perspective, the probability of “existence” is contingent upon whether a capsid structure can be produced via easily manageable assembly mechanisms (“ease of construction”). This is especially interesting since capsids with high 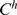 do not indicate larger size but just a more complicated design. E.g., 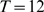 capsids, although smaller than 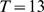 and 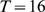 capsids, are vastly more complicated and under-represented in nature. Although our complexity-based rules imply a form of evolutionary pressure, other pressures will likely exist, whose effects might be overlaid to give a more intricate understanding of the available capsid distributions (e.g., geometrically simple 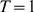 capsids, although low in 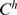, may be selected against due to restrictions of genomic size; see Fig. S3 in File S1).

### Understanding the Rule Breakers and Charting a Phase Diagram

#### Rule breakers

There are two major groups of 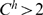 outliers/rule-breakers–the small (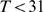) and large (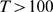) group–that display distinct characteristics. Markedly, most of the small rule-breakers possess an internal support/core of lipid or protein [19]–[21], or display unusually high number of protrusions and putative proteins associated with their capsomers [22]. These examples indicate that evolutionary constraints of a geometric nature placed upon isolated capsids may be overcome by employing “universal scaffolds” such as protein/bilayer cores and excessive auxiliary proteins useful in maintaining all distinct capsomer/hexamer shapes (Recently, another small rulebreaker not used in our study was also shown to have an internal membrane [23]). We predict that, generally, the amount of “extra subunit density” in the electron density of a capsid is directly related to 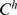.

**Figure 4:**
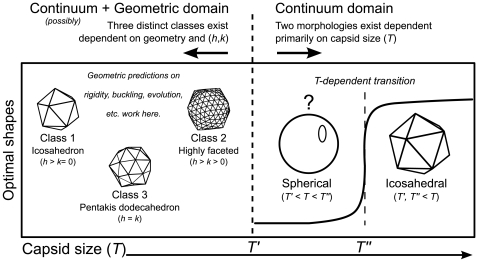
Spherical capsid phase diagram. We describe two specific capsid sizes that remain to be elucidated (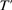 and 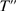; the diagram arbitrarily assumes that 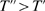). 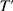 describes the limit of the geometric domain, beyond which our geometric assumptions and predictions may not hold. We expect that all capsid sizes greater than 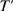 will be exclusively described by continuum elasticity. We also expect that, beyond 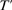 (i.e., in the purely continuum domain), the *Föppl-von Karman number* (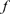) [27], [28] that dictates spherical vs. icosahedral morphology will depend primarily on 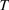, and so there will be a capsid size (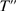) that demarcates the allowance for spherical and icosahedral morphologies in the purely continuum regime (the sigmoidal curve represents the dependence of 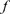 and hence morphology on 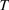). These assumptions consolidate all observed instances of spherical capsid morphology.

The remaining three (big) capsids [24], [25] that break our geometric rules possess thousands of subunits. This is interesting, since these capsids are possibly of large enough size that the “discreteness” (or geometric/molecular subunit nature) of the capsid shell has no influence on capsid morphology, which would allow for those capsids to be *exclusively* modeled as elastic shells [26]. This knowledge is helpful in constructing a proposed phase diagram for spherical capsids (Fig. 4).

#### Phase diagram

As described above, it is inevitable that, at a certain size or triangulation number (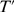 in Fig. 4), the capsid morphology will not be influenced by molecular/subunit/hexamer properties (where geometric relationships hold) [26], beyond which capsids may be modeled exclusively by continuum elasticity theory. Work using continuum elasticity has shown that only two capsid shapes must exist–spherical and icosahedral, and that the transition between them is demarcated by the capsid's *Föppl-von Karman number* (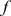) [27], [28], which is directly proportional to 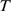 (especially if the size of the subunit is generally the same. This is because 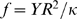 [27], where 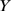 and 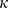 are bulk properties of the protein subunit, and 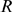 is the capsid's radius. If we assume that proteins, at an approximation, have similar size and bulk properties, then 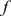 will be directly proportional to 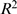, which is proportional to area, and therefore the number of protein subunits and hence to 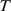). It is then interesting that the large (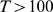) capsids are all icosahedral in shape, no matter what 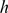-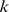 class they are present in. In our “phase diagram”, we also introduce a theoretical capsid size 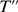 (Fig. 4 arbitrarily assumes that 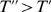) that differentiates between the sphere-icosahedron boundary predicted by continuum elasticity theory (the sigmoidal curve in Fig. 4 denotes the change in sphericity discussed before [27] that is dependent on 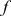 and hence capsid size, 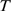).

The phase diagram brings to light a curious absence. So far, “hexamer complexity” was used to explain the elusiveness of certain capsid sizes (the 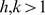 capsids peppered through size or 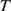-space). There is, however, a swath of the 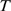-space (so far, between 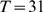 and 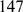) where no capsids, to our knowledge, have been reported. Beyond this 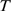 number swath, only purely icosahedron-shaped capsids have been observed. It will be interesting to see whether capsids from this region (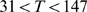) will be found in the future, and if so, what their will shapes be. Note that the diagram, although fitting all observed data, represents *one* situation where 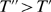 which does not need to be true (since we could also have 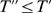, where “spherical capsids” in the continuum domain will never exist).

#### Continuum theory and the phase diagram

In continuum elasticity theory, 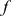 describes capsid morphology which ranges from completely spherical (for smaller 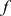) to completely faceted or icosahedral (for lager 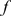). In the geometric sense, the sphericity of capsids in the class system decrease in the following manner: class 2 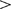 class 3 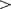 class 1. Within the geometric domain (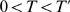), it is certain that shape is dictated by capsid class (described by 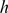-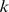) and not directly by capsid size (for example, 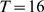 capsids are more faceted than 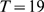 and 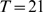 capsids; and 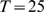 capsids are more faceted than 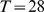 and 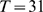 capsids). In light of this, if the continuum domain ranges to even the smallest capsids, we predict that 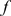 would increase non-monotonically (i.e. 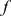 would fluctuate) through capsid size (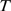-space) till 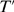, after which it will increase relatively smoothly and monotonically (w.r.t. 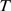) due to the absence of geometric (or 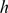-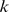 based) influences (Fig. 4). It will be interesting to find whether theoretical calculations are able to reiterate this trend, as it would then be possible to obtain an estimate for 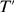.

### Further Implications

#### Classes, shapes and buckling

Because capsids from different classes display markedly different geometries, they are bound to display different physical properties. For example, since icosahedra and pentakis dodecahedra are geometrically rigid (this is a basic result of geometry), class 1 and class 3 capsids that employ such shapes should be unable to undergo buckling transitions (crucial virus life cycle events) [29], [30]. However, we expect class 2 capsids to be able to undergo such transitions due to their highly faceted (“harmonica like”) geometry, which allows for comfortable sampling of alternative structures. Also, class 1 and 3 capsids display a complete cage of endo angles spanning from pentamer to adjacent pentamer that serves as a frame to rigidify the structure. This is not the case for class 2 capsids, where endo propagations are prematurely terminated. Experimental work on one class 2 capsid, HK97 [29], [30], along with studies on capsid models (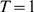 through 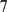 and 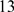) [11] lend credence to this hypothesis. Still, the existence of naturally buckling capsids of sizes other than 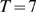 remains elusive.

#### 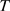-switching and pleomorphy

The periodic nature of capsid hexamer content (Fig. 3C) is also useful in understanding “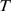-switching”: a process that permits *canonical* capsid subunits to more easily sample capsids containing similar hexamer shapes. This was shown to be true for a 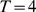 capsid subunit that, upon mutation, exclusively formed a range of class 1 capsids [31] that have similar hexamer shapes. This allows for a segue to understanding currently intractable and deadly pleomorphic viruses like ebola and arenaviruses. For example, from the above 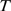-switching rule, the available diversity of an arenavirus (described by the observation of 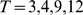 and 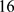 capsids in a single sample) [32] may only be explained if we assume that the biologically relevant form of the arena virus is the 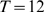 capsid (since it exclusively displays all hexamer species required for all the other listed capsid sizes excluding the flat hexamer, which allows us to assume that all other sizes are residual byproducts of inefficient 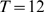 capsid assembly). Other predictions of this sort are easy to compile from Fig. 3C and remain to be completely developed, explored and validated.

#### Non-icosahedral capsids

Although the framework presented doesn't appear to readily explain non-isosahedral capsids (some are just “slightly” non-icosahedral, such as the natively prolate phi29 capsids [33], while others are wildly different in form, such as ebola with its natively filamentous shape), those capsids, like their icosahedral counterparts, also display capsomer sub-structures (for example phi29 capsids contain pentamers and hexamers, while there is evidence that filamentous ebola capsids may contain hexamers *as well as* octamers [34]). In light of this, the geometric constraints analogous to endo angles that affect capsomer shape may be useful in obtaining insights into non-icosahedral capsid morphology, behavior, and classification. It will be exciting to see whether incorporating the non-icosahedral capsids into an expanded capsid periodic table will be possible.

#### Ending note

Hexamer complexity (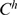) and the periodic table provide a framework that explains elusive evolutionary pressures on capsid design, 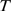-switching, mechanics (rigidity/maturation) and pleomorphy. We anticipate that many other features may be overlaid upon the schematic developed here, allowing for a comprehensive and systematic understanding of, first, spherical capsids and then virus capsids of varied geometries.

## Materials and Methods

### Geometric Models

The geometric models depicted in Fig. 2 were obtained by previous methods [11] that involve the realizations of graphs that define canonical capsids.

### Structural Databases Searched

Data paraphrased in Fig. 3A was compiled from 399 capsid structures culled to 119 representative structures obtained from the databases EMDB [17] and VIPER EMDB [35] for Electron Microscopy structures and VIPERdb [35] for X-ray structures, along with 4 structures that were not available in any of the databases (see Section L in File S1 for more details).

### Equation for Hexamer Complexity

An equation relating hexamer complexity (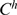) to capsid size (described by 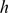 and 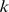) is derived in the Section J of File S1 and described as:
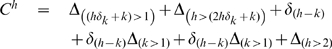
(1)Where
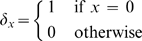
(2)and
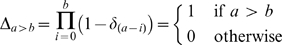
(3)


## Supporting Information

File S1 This supplementary document (1) reviews basic tenants/axioms developed from previous publications (Mannige and Brooks III, 2008 and 2009) that are used in the paper, (2) provides additional data on virus capsid abundances, (3) critically evaluates the validity of the results presented in the paper and (4) includes a list of viral capsids used in this study.
(0.38 MB PDF)
