# Supplementary material for: Periodic Table of Virus Capsids: Implications for Natural Selection and Design
Source: PLoS One. 2010 Mar 4;5(3):e9423. doi: 10.1371/journal.pone.0009423 (PMC2831995; doi:10.1371/journal.pone.0009423)
Supplement: File S1 — This supplementary document (1) reviews basic tenants/axioms developed from previous publications (Mannige and Brooks III, 2008 and 2009) that are used in the paper, (2) provides additional data on virus capsid abundances, (3) critically evaluates the validity of the results presented in the paper and (4) includes a list of viral capsids used in this study. (0.38 MB PDF) [file pone.0009423.s001.pdf]

SUPPORTING INFORMATION TO: *Periodic table of virus capsids: implications for natural selection and design*

Ranjan V. Mannige   Charles L. Brooks

January 27, 2010

Abstract

This text performs the following functions:

1. Defines endo angle propagation and termination (Section A, Fig. S1), hexamer complexity (Section C) and shape (Section D, Fig. S2).
2. Provides additional data on capsid abundances (Section F, Fig. S3) further indicating the utility of hexamer complexity (Section C) and the periodic table (Fig. 3C) in explaining evolutionary pressures (Section E).
3. Critically evaluates the validity of our results (Section G).
4. Provides a formalism for calculating hexamer complexity (Sections H-K).
5. And finally provides the list of all capsid structures used in validating the theory (Section L).

A Endo angle propagation and termination rules.

A result of subunit quasi-equivalence introduced in Ref. [1] (and discussed in Fig. 1B) and the trapezoidal subunit shape (a ubiquitous capsid subunit shape [2] present in viruses infecting all domains of life [4]) is that the inter-subunit angles (subunit-subunit dihedral angles) originating from the

pentamer (*endo angles*, introduced earlier [3]) must propagate through the adjacent hexameric lattice (depicted as arrows in Fig. 1B) in what we call *endo angle propagation.* Although endo angle propagation has been shown to affect neighboring hexamer shape within the natural canonical capsid [2], the interaction/interference of multiple propagations in the confines of a capsid has not been completely investigated and is discussed in Fig S1.

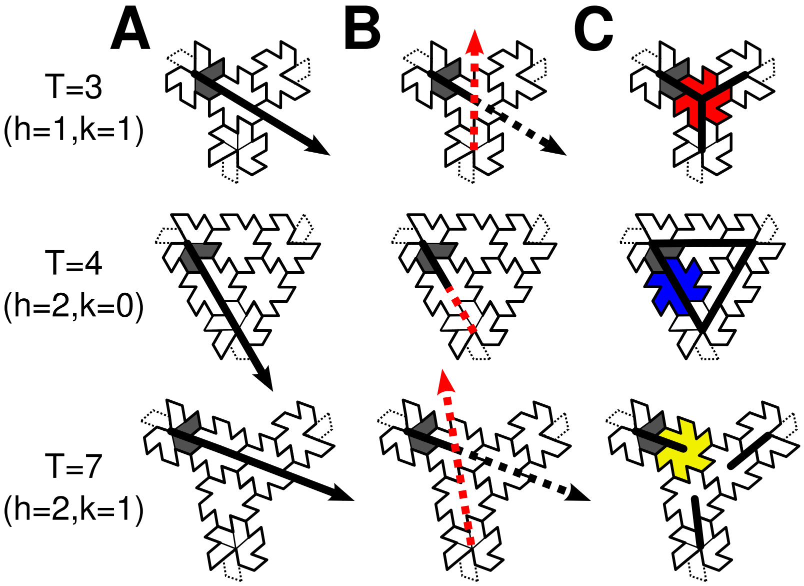

Figure S1: We define *endo angle rules* for the three smallest capsids possessing hexamers ($T = 3, 4, 7$) within a "face" (a triangular facet containing hexamers and three adjacent pentamers). An endo angle (black ray) propagating from the shaded subunit-subunit interface belonging to a pentamer (**A**) is challenged and terminated by another endo angle (**B**, red dotted ray) propagated from a neighboring pentamer, not completely visible for $T = 4$), resulting in hexamer shapes and capsid endo angle features (**C**) that are h and k specific. In particular the differences in h-k relationships ensure hexamers of distinct shapes per capsid size (distinctly colored).

B Canonical vs. noncanonical capsids

All our specific predictions are directed towards canonical capsids where subunits (within any given capsid) are tilable and nearly-invariant in shape[2].

This is because the consequence of introducing/imposing curvature into the shell is conveniently imposed as endo angle propagations[3], which then allows for hexamer shapes to be precisely characterized (Section C). However, that our predictions apply to all structurally characterized spherical capsids indicate parallel constraints applied to noncanonical capsid hexamers. It will be interesting to see the differences and similarities between the constraints acting on canonical and noncanonical capsids.

C Defining hexamer complexity C^h

Hexamer complexity C^h is the minimal number of distinct hexamer shapes that a canonical capsid [2] of specific size (defined by h, k or T) contains. The possible hexamer shapes that a canonical capsid may possess are shown in Fig. S2B (derived by inspecting Fig. 2 and assuming the working of endo angle propagation and termination rules in Fig. S1).

D Counting hexamer shapes

Previously, we showed that different arrangements of **e**ndo dihedral angles (designated "**e**") among non-endo, or e**x**o angles (designated "**x**") in a hexamer define distinct hexamer shapes [3]. This assumption has been shown to be true for those natural canonical capsids that have afforded investigation [3]; specifically, we showed that the smallest capsids from each class ($T = 3, 4, 7$) possess distinct hexamer shapes, named in accordance with the hexamer coloring in Figs. 2 and 3: red ($\mathbf{e}x\mathbf{e}x\mathbf{e}x$; "ruffled"), blue ($\mathbf{e}xx\mathbf{e}xx$; "wing shaped"), and yellow ($\mathbf{e}xxxxx$, "single-pucker") hexamer shapes respectively [3]. These capsids possess the lowest C^h of one.

Larger capsids increase in C^h due to the requirement of additional hexamer shapes colored in Fig. 2 as green ($xxxxxx$; "flat"[1]) and cyan ($\mathbf{e}'xx\mathbf{e}'xx$, shaped as an "inverse wing" possessing *inverse endo angles* $\mathbf{e}'$ whose acute angles face outward).

[1]In the $h > k = 1$ capsids, the green hexamer is not perfectly flat, but will tend towards possessing identical dihedral angles, which, for a hexamer, optimally would result in generally flat hexamers.

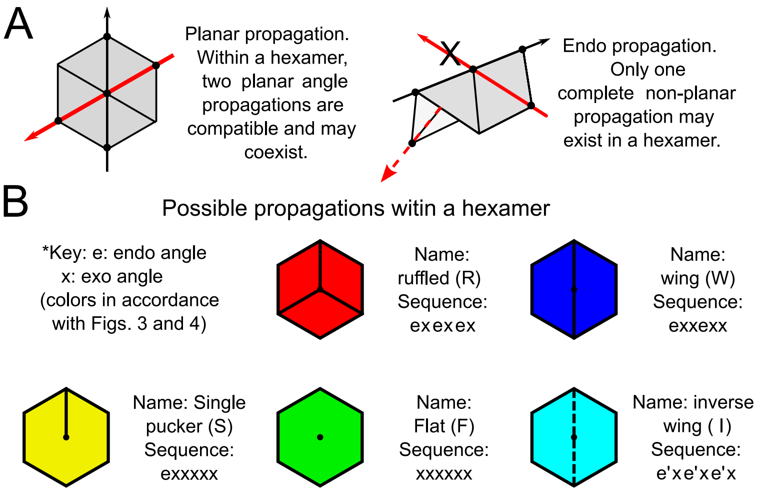

Figure S2: **Hexamer shapes available to capsids**. (**A**), although planar endo angle constraints are able to freely propagate within hexamers (left), only one *complete* non-planar (or “endo”) angle constraint/propagation may be present within a hexamer (collinear propagations not included). If two non-linear/non-parallel propagations meet, one must terminate at that meeting point, which means that multiple non-linear endo angles may exist within a single hexamer only if *terminated* at its center. (**B**), Possible arrangements of terminal endo angles (reflecting possible hexamer shapes) are listed (endo angles are represented as lines in the hexamer diagrams and as **e** in the hexamer angle sequence; **e**′ represents an inverse endo angle). The hexamers are colored in accordance with the Fig. 2.

E Capsids with low C^h are preferred

From Fig. S3, we can surmise that, for the range of T numbers observed ($T = 1...219$ and for a more conservative/truncated range, $T = 1...31$), capsids with lower C^h appear to be preferred as evidenced by a shift to lower C^h distributions in observed versus expected capsid distributions. Table S1 lists the first twelve capsid sizes (T) by class; those sizes displaying $C^h > 2$ are indicated by boldface.

A major difference between the red and black graphs in Fig. S3 comes in the behavior in abundances of expected $C^h = 3$ capsids, that mostly belong to the $h > k > 1$ regime. Specifically, as we increase from the $(n-1)^{th}$ period to n^{th} period in the periodic table, class 1 (where C^h mostly equals 2) and class 3

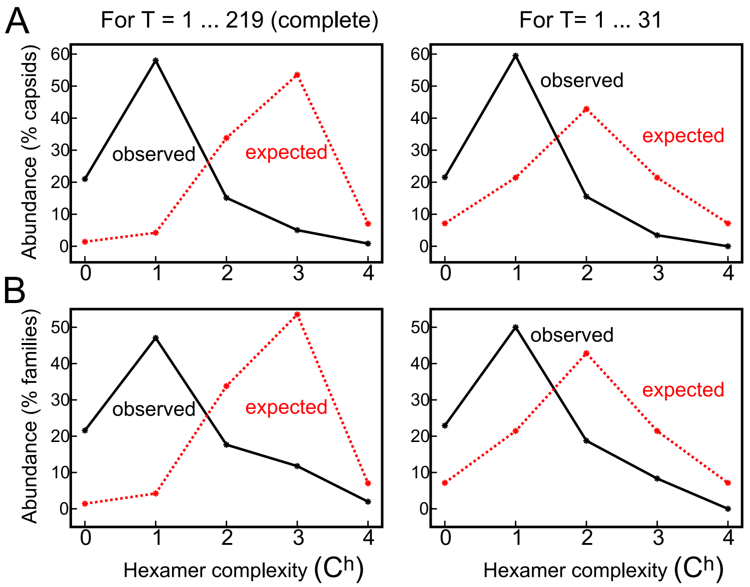

Figure S3: **Capsids tend to prefer lower C^h than expected**. Plotted in each graph is C^h versus observed (solid, black lines) and expected abundances (dotted, red lines) obtained from 119 capsids (**A**) and 52 family entries (**B**) each shown for the complete available capsid size range ($T = 1...219$; left) and a truncated range (right). The expected dataset assumed a uniform size(T)-distribution for capsids in the displayed T-range. Family entries represent individual families, except for families displaying more than one capsid size, which were split to maintain one C^h per family entry.

entries (where C^h mostly equals 4) increase by 1, while the class 2 entries (where C^h mostly equals 3) increase in a more-or-less arithmetic progression by $(n-1)$ (evident in Fig 3C in the triangular shape of the class 2 group versus linear shapes of class 1 and class 3 groups respectively).

F Observed capsid abundance $\propto 1/C^h$

Finally, excepting $C^h = 0$ capsids (i.e., capsids that contain no hexamers, or $T = 1$ capsids), there is an inverse relationship between C^h and observed capsid abundance (black lines in Fig. S3). The low observed abundance for $C^h = 0$ capsids is expected, given that most virus families with true $C^h = 0$ appear to be too small to accommodate enough genomic material to infect

Table S1: The distribution of capsid sizes into the three morphological classes described by the relationship between the capsid's h and k. The percentage abundance ($A(\%)$) of capsids in the three classes were obtained from a collection of 118 non-redundant capsids belonging to 39 diverse capsid families.

Class	h-k	$A(\%)$	Triangulation (T) number series
1	$h > k = 0$	33.9	1 4 9 16 25 ...
2	$h > k > 0$	22.8	7 13 **19** 21 ...
3	$h = k$	43.2	3 **12** **27** ...

as a primary source (therefore, most true $T = 1$ capsids belong to "satellite viruses" that are only able to infect hosts preinfected by a primary infector, presumably since those virus capsids provide insufficient volume to contain an independent infectious genome). Here, the additional/stronger evolutionary impediment appears to be a lower bounded genome size preference (i.e., a non-geometric preference imposing a constraint of $C^h > 0$ may be overlaid with the inverse C^h rule to obtain the observed or black graphs in Fig. S3).

G Is there a data-collection bias?

Here, we address the question: are our findings a result of a basic inability to sample structures of large C^h, or does the data truly reflect our predictions?

Fig. S3 (reflecting the rest of our data) was produced from a compilation of capsids obtained from (1) X-ray crystallography, whose prowess lies in obtaining high resolution capsid 3D structures of "small" sizes (e.g., $T =$ 1...25), and (2) electron microscopy, where large capsids do not disallow the elucidation of capsid size or T number (which can be obtained from simple electron micrographs, if not by 3D capsid reconstructions). Consequently, we argue that if observable to a structural virologist, any new capsid of any size would not be far from finding a public domain home (thereby finding its way in our graphs). Thus we argue that our observed data does not reflect discrepancies in data collection as much as it lends credence to our geometric predictions.

Furthermore, if capsid collection were to be size constrained, it would sill not matter so much, since our existence rules are not size dependant as much as h, k dependant (e.g., although smaller than $T = 25$, the $T = 19$ capsid is expected to be higher in hexamer complexity and therefore lower

in abundance, which is the case).

H Basic definitions

The Kronecker delta function (δ_x) is quite integral to our future formalisms, and is therefore introduced here as a special topic. Specifically, δ_x (or $\delta_{x,0}$) is an algorithm, that outputs 1 if x=0 and 0 otherwise, i.e.,

$$\delta_x = \begin{cases} 1 & \text{if } x = 0 \\ 0 & \text{otherwise} \end{cases} \tag{1}$$

We can represent this algorithm by the limits

$$\delta_x = \lim_{\alpha \to \infty} \frac{1}{e^{\alpha x^2}} \tag{2}$$

or

$$\delta_x = \lim_{\alpha \to \infty} \frac{2e^{\alpha x}}{1 + e^{2\alpha x}} \tag{3}$$

which may be used later on.

We also utilize a convenient equation that produces a binary output after comparing two non-negative integers a and b:

$$\Delta_{a>b} = \prod_{i=0}^{b} \left(1 - \delta_{(a-i)}\right) = \begin{cases} 1 & \text{if } a > b \\ 0 & \text{otherwise} \end{cases} \tag{4}$$

Some basic definitions:

$$\delta_a \left(1 - \delta_a\right) = \begin{cases} 1 \times (1 - 1) & \text{if } a = 0 \\ 0 \times (1 - 0) & \text{otherwise} \end{cases} \tag{5}$$

i.e., for all cases,

$$\delta_a \left(1 - \delta_a\right) = 0 \tag{6}$$

Also, it follows that

$$a\delta_a = 0 \tag{7}$$

and

$$\begin{aligned} \delta_a \Delta_{a>b} &= \delta_a(1 - \delta_a) \times [(1 - \delta_{a-1})...(1 - \delta_{a-b})] \\ &= 0 \times [(1 - \delta_{a-1})...(1 - \delta_{a-b})] \\ &= 0 \end{aligned} \tag{8}$$

I Obtaining endo propagation length $\phi_{h,k}$

Definition of endo angle propagation length $\phi_{h,k}$. It is the distance (in capsomers, including the originating pentameric angle) that the endo angle is allowed to propagate from a pentamer into the hexamers before being intercepted (or terminated). Please refer to Fig. S1 for a review of the endo angle propagation and termination rules.

From Fig. S1 (and corroborated in Fig. 2), we can obtain the endo angle propagation length for a capsid of size h, k:

$$\phi_{h,k} = \begin{cases} h & \text{for class 1, i.e., if } k = 0 \\ k & \text{otherwise, i.e., if } k \neq 0 \end{cases} \tag{9}$$

which can be described as

$$\phi_{h,k} = (h\delta_k + k) = \begin{cases} h & \text{if } k = 0 \\ k & \text{otherwise} \end{cases} \tag{10}$$

J Obtaining C^h from h, k and $\phi_{h,k}$

Here, we obtain a mathematical/algorithmic expression for C^h. We can treat the hexamer complexity C^h as a sum of its components C^h_X, where X may be one of the five distinct hexamer shapes (i.e., $X \in (W, R, S, F, I)$), and $C^h_X = 1$ only if the hexamer shape "X" exists within the capsid. We now attempt to obtain the C^h components for each hexamer shape.

Wing shaped (W). The presence of two linear adjacent endo angles within a hexamer automatically indicate that wing shaped hexamers must exist within the capsid, since the only hexamer that can accommodate two linear angles is the winged shape of profile **e**xx**e**xx [3] (we define a *linearly adjacent angle set* as a set of two angles within the hexamer of position i and $i + 3$, where $i = i + 6$, indicating the cyclic nature of the angles). Therefore, we will expect wing shaped hexamers when $\phi_{h,k} > 1$. So, the hexamer complexity contribution by the presence of a wing shaped hexamer will be

$$C^h_W = \Delta_{((h\delta_k+k)>1)} \tag{11}$$

Single pucker shaped (S). We can define the closest distance (in capsomer units) between two adjacent pentamers ($P_{h,k}$) as

$$P_{h,k} = (h + k) \tag{12}$$

Table S2: Hexamer complexity, C^h from Eqn. 18.

T	h	k	C^h_W	$+\ C^h_S$	$+\ C^h_R$	$+\ C^h_I$	$+\ C^h_F$	$= C^h$
1	1	0	0	0	0	0	0	0
3	1	1	0	0	1	0	0	1
4	2	0	1	0	0	0	0	1
7	2	1	0	1	0	0	0	1
9	3	0	1	0	0	0	1	2
12	2	2	1	0	1	1	0	3
13	3	1	0	1	0	0	1	2
16	4	0	1	0	0	0	1	2
19	3	2	1	1	0	0	1	3
21	4	1	0	1	0	0	1	2
25	5	0	1	0	0	0	1	2
27	3	3	1	0	1	1	1	4
28	4	2	1	1	0	0	1	3
31	5	1	0	1	0	0	1	2
36	6	0	1	0	0	0	1	2

Which is an interesting value, since it is also the maximum number of capsomers that the endo angle can propagate through, i.e.,

$$P_{h,k} \geq \phi_{h,k} \tag{13}$$

We can also show that if $\phi_{h,k} \geq P_{h,k}/2$, then the endo angles will form a complete/unbroken cage around the capsid (which is seen in classes 1 and 3). However, if we do not have "complete propagation", then we are guaranteed the existence of a single pucker hexamer, i.e.,

$$\begin{aligned} C^h_S &= \Delta_{\left(P_{h,k} > 2\phi_{h,k}\right)} \\ &= \Delta_{((h+k)>(2h\delta_k+2k))} \\ &= \Delta_{(h>(2h\delta_k+k))} \end{aligned} \tag{14}$$

Ruffle shaped. We also know that if $h = k$ (class 3) then $P_{h,k} = 2\phi_{h,k}$ (because if $h = k$ then $2\phi_{h,k} = 2h = h + k = P_{h,k}/2$) and three adjacent endo angles will terminate at the central hexamer causing the presence a hexamer of $\mathbf{e}x\mathbf{e}x\mathbf{e}x$ profile and of ruffled shape, so

$$C^h_R = \delta_{(h-k)} \tag{15}$$

Inverse-wing shaped. We know that the ruffled $\mathbf{e}x\mathbf{e}x\mathbf{e}x$ profile is rigid [3], so even the exo (x) to must remain constrained. Since this dihedral's acute angle faces the outside portion of the capsid, we call this special angle the inverse endo (e') angle. Since inverse endo angles are constrained, they must propagate between any two ruffled hexamers, resulting in the formation of a special inverse-wing shape in large enough capsids ($h, k > 1$) containing ruffled hexamers ($h = k$), i.e., we have

$$C^h_I = C^h_R \Delta_{(h>1)} = \delta_{(h-k)}\Delta_{(h>1)} = \delta_{(h-k)}\Delta_{(k>1)} \quad (16)$$

Flat shaped. Finally, we know that a capsid of large enough size ($h > 2$) irrespective of class, must possess hexamers that are generally unaffected by endo angle constraints which are therefore generally flat, so

$$C^h_F = \Delta_{(h>2)} \quad (17)$$

Combining the above C^h components, our resulting relationship for hexamer complexity will be

$$\begin{aligned} C^h &= C^h_W + C^h_S + C^h_R + C^h_I + C^h_F \\ &= \Delta_{((h\delta_k+k)>1)} + \Delta_{(h>(2h\delta_k+k))} + \delta_{(h-k)} \\ &\quad + \delta_{(h-k)}\Delta_{(k>1)} + \delta_{(h-k)}\Delta_{(k>1)} + \Delta_{(h>2)} \end{aligned} \quad (18)$$

K The number of hexamers N_X

We list the number of hexamers N_X per hexamer type X:

$$N_W = \left(\frac{60(h\delta_k + k)}{1 + \delta_k}\right) C^h_W \quad (19)$$

$$N_S = 60C^h_S \quad (20)$$

$$N_R = 20C^h_R \quad (21)$$

$$N_I = (h-1)\, C^h_I \quad (22)$$

$$N_F = \left(10(T-1) - \sum_{X\in[W,S,R,I]} N_X\right) C^h_F \quad (23)$$

The list (Section L) of all virus capsids used in the abundancy analysis is available in the file *MannigeBrooks_SI_b.xls*.

References

[1] D. L. D. Caspar and A. Klug. Physical principles in the construction of regular viruses. *Cold Spring Harbor Symp.*, 27:1–24, 1962.

[2] R. Mannige and C. Brooks III. Tilable nature of virus capsids and the role of topological constraints in natural capsid design. *Phys. Rev. E*, 77(5):051902, 2008.

[3] R. Mannige and C. Brooks III. Geometric considerations in virus capsid size specificity, auxiliary requirements, and buckling. *Proc. Natl. Acad. Sci. USA.*, 106(21):8531–8536, 2009.

[4] G. Rice, L. Tang, K. Stedman, F. Roberto, J. Spuhler, E. Gillitzer, J. Johnson, T. Douglas, and M. Young. The structure of a thermophilic archaeal virus shows a double-stranded dna viral capsid type that spans all domains of life. *Proc. Natl. Acad. Sci. USA*, 101(20):7716–7720, 2004.

Data used in the manuscript: ***Periodic table of virus capsids: implications for natural selection and design***

by Mannige and Brooks

Collected by Ranjan Mannige, Brooks Lab, Scripps Research Institute/Univ. of Michigan at Ann Arbor

NUMBER OF FAMILIES STUDIED: 36 known, and 4 capsids with unknown classification.

Please email corresponding authors for most updated spreadsheet.

Sources:

DB Source	Model Type	Website	# structures	Date updated
**EMDB**	EM/CryoEM	http://www.ebi.ac.uk/msd-srv	95	07/16/08
**VIPER EMDB**	EM/CryoEM	http://viperdb.scripps.edu/EN	60	07/16/08
**VIPERdb**	crystal st.	http://viperdb.scripps.edu/	211	07/16/08
**VIPERdb**	pdb “models”	http://viperdb.scripps.edu/	29	07/16/08
**OTHER**	EM/CryoEM	-	4	07/16/08
**TOTAL Entries:**			**399**	

Summary:

TOTAL capsids	Rulebreakers	Families	Class I	Class II	Class III
119	7	51[#]	40	27	52

\# Capsids from families with two or more sizes are distinctly annotated.

All entries:

---

p: not in searched databases

(m): Model

(l): low resolution

T	h,k	EMDB Ids	Viper EMDB IDS	VIPERdb	Family	Name
1	1,0	1178, 1179	em_1178	2c9g, 2c9f, 2c6s, 1x9t, 1x9p	Adenoviridae	Adenovirus dodecahedron
25p	5,0	1272, 1016, 1112, 1464	em_1111, em_1113	2bld(m)	Adenoviridae	Human Adenovirus
25p	5,0	1462, 1463	-	-	Adenoviridae	Canine Adenovirus (serotpe 2)
25p	5,0	1489, 1490	-	-	Adenoviridae	Human adenovirus type 5
1	1,0	1237	-	1wcd	Birnaviridae	IBDV Subviral Particle
13l	3,1	1118, 1238,* 1239	em_1115	1wce	Birnaviridae	Infectious Bursal Disease Virus
1	1,0	-	em_3bmv	1yc6	Bromoviridae	Brome Mosaic Virus
3	1,1	-	em_1bmv, em_2bmv	1js9	Bromoviridae	Brome Mosaic Virus
3	1,1	-	-	1laj	Bromoviridae	Tomato Aspermy Virus
3	1,1	-	em_1cwp, em_2cwp	1za7	Bromoviridae	Cowpea Chlorotic Mottle Virus
3	1,1	-	-	1f15	Bromoviridae	Cucumber Mosaic Virus
12(p?)	2,2	**p**	-	-	Bunyaviridae	Uukuniemi virus
3	1,1	-	-	1ihm	Caliciviridae	Norwalk Virus
3	1,1	-	-	2gh8	Caliciviridae	A native Calicivirus (genus: vesivirus)
7	2,1	-	em_1cam	-	Caulimoviridae	Cauliflower Mosaic Virus
3p	1,1	-	-	1a6c	Comoviridae	Tobacco Ringspot Virus
3p	1,1	-	-	1b35	Comoviridae	Cricket Paralysis Virus
3p	1,1	-	-	1bmv, 1pgl, 1pgw	Comoviridae	Bean Pod Mottle Virus
3p	1,1	-	em_1cmv, em_2cmv, em_3cmv, em_4cmv	1ny7	Comoviridae	Cowpea Mosaic Virus (components)
3p	1,1	1512	-	-	Comoviridae	Blackcurrant reversion nepovirus
21pd	4,1	1083, 1084, 1085	em_1082	-	Corticoviridae	PM2
1 (p2)	1,0	1300	-	-	Cystoviridae	Bacteriophage Phi8 core
1 (p2)	1,0	1500, 1501, 1502, 1503	-	-	Cystoviridae	Bacteriophage phi6 procapsid
13l	3,1	1206, 1207, 1301	-	-	Cystoviridae	Bacteriophage phi6
13l	3,1	1299	-	-	Cystoviridae	Bacteriophage Phi8 virion
3	1,1	1166, 1167	em_1166	1k4r(l), 1thd(m), 1p58(m), 1tge(m), 1n6g(m)	Flaviviridae	Dengue virus
3	1,1	1418	-	-	Flaviviridae	Dengue 2 virus
3	1,1	-	-	1na4(m)	Flaviviridae	Yellow Fever virus
3	1,1	1234	-	2of6(m)	Flaviviridae	West nile virus
4	2,0	1399, 1400, 1401, 1402, 1403, 1404, 1405, 1406, 1407, 1408	-	1qgt, 2g34, 2g33	Hepadnavirus	Hepatitis B virus
16	4,0	1354	-	-	Herpesviridae	HSV-1 C-capsids
147	7,7	**p**	-	-	Iridoviridae	chilo iridescent virus (CIV)
3	1,1	-	-	1gav	Leviviridae	Bacteriophage GA Protein Capsid

3	1,1	-	-	1frs, 1fr5	Leviviridae	Bacteriophage FR
3	1,1	-	-	1dwn	Leviviridae	Bacteriophage PP7
3	1,1	1431, 1432, 1433	-	1aq3, 1aq4, 1bms,1dzs, 1dzs, 1e7x, 1gkv, 1gkw, 1kuo, 1mst, 1mva, 1mvb, 1u1y, 1zdh, 1zdi, 1zdj, 1zdk, 2b2e, 2b2g, 2bu1, 2c4y, 2c4z, 2c50, 2c51, 2ms2, 5msf, 6msf, 7msf	Leviviridae	Bacteriophage MS2
3	1,1	-	-	1zse, 2b2d, 1qbe	Leviviridae	Bacteriophage Q beta
3	1,1	-	em_1byd	-	Luteoviridae	Barley Yellow Dwarf Virus
1	1,0	-	-	1gff	Microviridae	Bacteriophage G4
1	1,0	-	-	1m0f(m), 1m06	Microviridae	Bacteriophage alpha3
1	1,0	-	-	1kvp(m)	Microviridae	Spiroplasma Virus, SPV4
1	1,0	-	em_1pxa, em_1pxb	1cd3, 1al0, 1rb8, 2bpa	Microviridae	Bacteriophage phix174
3	1,1	-	em_1fhv	-	Nodaviridae	Flock House Virus
3	1,1	-	-	2bbv	Nodaviridae	Black Beetle Virus
3	1,1	-	em_1f8v	1f8v	Nodaviridae	Pariacoto Virus
3	1,1	-	-	1nov	Nodaviridae	Nodamura Virus
1	1,0	-	-	1dzl	Papillomaviridae	Human Papilloma Virus 16
7d	1,2	-	-	1l0t(m)	Papillomaviridae	Human Papilloma Virus
7d	1,2	-	em_1bpv	-	Papillomaviridae	Bovine Papilloma Virus Type 1
1 (2p)	1,0	1459	-	-	Partitiviridae	Partitivirus (PsV-S)
1	1,0	-	-	1c8d, 1c8g, 1c8f, 1c8e	Parvoviridae	Canine Panleukopenia virus
1	1,0	-	em_1gmd	1dnv	Parvoviridae	Galleria Mellonella Densovirus
1	1,0	-	-	1k3v	Parvoviridae	Porcine Parvovirus
1	1,0	-	-	1fpv	Parvoviridae	Feline Panleukopenia virus
1	1,0	-	em_2cpv	1c8h, 1ijs, 4dpv, 2cas, 1p5w, 1p5y	Parvoviridae	Canine Parvo Virus
1	1,0	-	-	2g8g, 2qa0, 1lp3	Parvoviridae	Adeno-Associated virus
1	1,0	1326	-	1mvm. 1z14	Parvoviridae	Minute Virus of Mice strain I
1	1,0	1466, 1467, 1468	em_1b19, em_2b19	1s58	Parvoviridae	Human Parvovirus B19
169d	7,8	-	em_1pbc	1m4x(m)	Phycodnaviridae	Paramecium Bursaria Chlorella Virus
219d	7,10	**p**	-	-	Phycodnaviridae	Marine Algal Virus PpV01
3p	1,1	-	-	1tmf, 1tme	Picornaviridae	Theiler Murine Encephalomyelitis
3p	1,1	-	-	svv*	Picornaviridae	Senecavirus
3p	1,1	-	-	1zba, 1zbe, 1qqp, 1bbt, 1fod, 1qgc(m), 1fmd	Picornaviridae	Foot-and Mouth Disease Virus
3p	1,1	1133, 1137, 1144	em_1136	1piv, 1dgi(m), 1nn8(m), 1hxs, 1asj, 1ar7, 1ar6, 1ar8, 1ar9, 1al2, 1vbd, 1po2, 1po1, 1vbc, 1vba, 1vbb, 1vbe, 1xyr(m), 1eah, 1pvc, 1pov, 2plv	Picornaviridae	Poliovirus
3p	1,1	1057, 1058, 1182	em_1183	1mqt, 1oop	Picornaviridae	Swine Vesicular Disease virus
3p	1,1	1057, 1058, 1182	em_1183	2c8i(m), 1upn(m), 1ev1, 1h8t, 1m11(m)	Picornaviridae	Echovirus
3p	1,1	1411, 1114	em_1114	1cov, 1jew, 1z7z, 1z7s, 1d4m	Picornaviridae	Coxsackievirus
3p	1,1	-	em_1049, em_1hrb, em_1hrc, em_2hra, em_2hrb	2hwb, 2hwc, 2hwd, 2hwe, 2hwf, 1d3i(m), 1d3e(m), 1k5m, 1r1a, 1rvf, 1hrv, 1vrh, 1r09, 1ayn, 1aym, 1qju, 1qjy, 1qjx, 1v9u, 1rhi, 1fpn, 1c8m, 1ruf, 1ruc, 1rud, 1rug, 1ruh, 1rui, 1ruj, 1rue, 1r08, 2r04, 2r06, 2r07, 2rm2, 2rr1, 2rs1, 2rs3, 2rs5, 1hri, 1na1, 1ncq, 1nd3, 1nd2, 1ncr, 4rhv, 1rmu, 2rmu	Picornaviridae	Human Rhinovirus
3p	1,1	-	-	1mec, 2mev	Picornaviridae	Mengovirus
3p	1,1	-	-	1bev	Picornaviridae	Bovine Enterovirus VG-5-27
3	1,1	-	em_1116, em_1120	-	Podoviridae	isometric phi29 particle

4	2,0	1281	-	-	Podoviridae	isometric phi29 particle (rossmann)
7l	2,1	1339	-	-	Podoviridae	Cyanophage Syn5
7l	2,1	1321	-	-	Podoviridae	Phage T7 prohead
7l	2,1	1101	-	-	Podoviridae	Bacteriophage p22
7l	2,1	1509	-	-	Podoviridae	Bacteriophage N4
7d	1,2	-	-	1sid, 1sie	Polyomaviridae	Murine Polyoma virus
7d	1,2	-	em_1sva	-	Polyomaviridae	Simian Virus 40
1	3,1	-	em_1reo	1ej6	Reoviridae	Reovirus Core
13l	3,1	1165, 1508	-	-	Reoviridae	Cytoplasmic Polyhedrosis Virus
13l	3,1	-	em_1177	-	Reoviridae	Broadhaven virus
13l	3,1	-	-	2btv	Reoviridae	Bluetongue virus (BTV)
13l	3,1	1532	-	-	Reoviridae	Grass Carp Reovirus Core and Virion
13l	3,1	1060, 1375, 1377, 1379, 1381, 1383, 1385, 1387, 1389	-	1uf2	Reoviridae	Rice Dwarf Virus
13l	3,1	1460	-	-	Reoviridae	Bovine rotavirus DLP
19	3,2	p	-	-	Reoviridae	“Misformed” rotavirus (J. Virol., 82:2844)
1	1,0	-	-	1a34	Satellites	Satellite Tobacco Mosaic virus
1	1,0	-	-	1stm	Satellites	Satellite Panicum Mosaic virus
1	1,0	-	-	2buk	Satellites	Satellite Tobacco Necrosis virus
7l	2,1	-	-	2frp, 2fte(m), 2ft1, 2fs3, 2fsy, 1if0(m), 1ohg	Siphoviridae	Bacteriophage HK97
7l	2,1	1507	-	-	Siphoviridae	Lambda procapsid
1	1,0	-	-	1x36, 1vb4, 1vak, 1vb2	Sobemoviridae	Sesbania Mosaic virus mutant (T=1)
3	1,1	-	-	4sbv	Sobemoviridae	Southern Bean Mosaic virus
3	1,1	-	-	1smv, 1x35, 1x33	Sobemoviridae	Sesbania Mosaic virus
3	1,1	-	-	2izw	Sobemoviridae	Ryegrass Mottle virus
3	1,1	-	-	1f2n	Sobemoviridae	Rice Yellow Mottle virus
3	1,1	-	-	1ng0	Sobemoviridae	Cocksfoot Mottle virus
25p	5,0	1012, 1013, 1014	em_1011	1gw7(m), 1hb5(m), 1hb7(m), 1gw8(m), 1hb9(m), 1w8x9	Tectiviridae	Bacteriophage PRD1
25p	5,0	1123, 1124	em_1124	-	Tectiviridae	Bacteriophage Bam35
4	2,0	-	em_1prv	-	Tetraviridae	Providence Virus
4	2,0	-	em_1nbv, em_1nwv, em_2nwv, em_3nwv, em_4nwv, em_5nwv	1ohf	Tetraviridae	Nudaurelia Beta Capensis Virus
3	1,1	-	-	2e0z	Thermococcaceae	VLP from Pyrococcus furiosus
4	2,0	1437	em_1121, em_1sin	-	Togaviridae	Sindbis virus (alphavirus)
4	2,0	-	em_1rrv, em_2rrv	-	Togaviridae	Ross River Virus
4	2,0	-	em_1aur	-	Togaviridae	Aura Virus
4	2,0	1018	em_1015	1dyl(m), 1ld4(m)	Togaviridae	Semliki Forest Virus
3	1,1	-	-	2tbv	Tombusviridae	Tomato Bushy Stunt virus
3	1,1	-	-	1opo	Tombusviridae	Carnation Mottle virus
3	1,1	-	-	1tnv, 1c8n	Tombusviridae	Tobacco Necrosis virus
1	1,0	-	em_1m1c	-	Totiviridae	L-A Virus
1	1,0	-	em_1umv	-	Totiviridae	Ustilago Maydis Virus H1
3	1,1	-	-	1ddl	Tymoviridae	Desmodium Yellow Mottle tymovirus
3	1,1	-	-	2fz2, 1w39, 2fz1, 1auy	Tymoviridae	Turnip Yellow Mosaic virus
3	1,1	-	-	1e57, 1qjz	Tymoviridae	Physalis Mottle virus
28	4,2	1350, 1353, 1351, 1352	-	-	Unknown1	Haloarchaeal virus SH1
31	5,1	-	em_1ynv	-	Unknown2	Sulfolobus Turretted Icosahedral Virus
7l	2,1	5003	-	-	Unknown3	Bacteriophage epsilon15
3p	1,1	1150, 1153	em_1154	-	Unknown4	Kelp fly virus
27	2,2	1392	-	-	Caudovirales	phiKZ
